# Analysis of Selenoprotein Expression in Response to Dietary Selenium Deficiency During Pregnancy Indicates Tissue Specific Differential Expression in Mothers and Sex Specific Changes in the Fetus and Offspring

**DOI:** 10.3390/ijms21062210

**Published:** 2020-03-23

**Authors:** Pierre Hofstee, James S.M. Cuffe, Anthony V. Perkins

**Affiliations:** 1 School of Medical Science, Menzies Health Institute Queensland, Griffith University Gold, Coast Campus, Southport, QLD 4215, Australia; p.hofstee@griffith.edu.au; 2 The School of Biomedical Sciences, The University of Queensland, St Lucia, QLD 4072, Australia

**Keywords:** pregnancy, selenium, nutrients, gene expression, DOHaD, reproduction

## Abstract

The human selenoproteome is comprised of ~25 genes, which incorporate selenium, in the form of selenocysteine, into their structure. Since it is well known that selenium is important to maternal health and foetal development during pregnancy, this study aimed at defining the impact of selenium deficiency on maternal, placental, foetal and offspring selenoprotein gene expression. Female *C57BL/6* mice were randomly allocated to control (>190 μg/kg) or low selenium (<50 μg/kg) diets four weeks prior to mating and throughout gestation. At embryonic day (E)18.5, pregnant mice were sacrificed followed by collection of maternal and foetal tissues. A subset of mice littered down, and offspring were monitored from postnatal day (PN) 8, weaned at PN24 and sacrificed at PN180, followed by tissue collection. Following RNA extraction, the expression of 14 selenoproteins was assessed with qPCR in liver, kidneys, muscle and placenta. Selenium deficiency downregulated expression (*P*_trt_ < 0.05) of many selenoproteins in maternal tissues and the placenta. However, foetal selenoprotein expression was upregulated (*P*_trt_ < 0.05) in all tissues, especially the kidneys. This was not reflected at PN180; however, a sexually dimorphic relationship in selenoprotein expression was observed in offspring. This study demonstrates the selenoproteome is sensitive to dietary selenium levels, which may be exacerbated by pregnancy. We concluded that transcriptional regulation of selenoproteins is complex and multifaceted, with expression exhibiting tissue-, age- and sex-specificities.

## 1. Introduction

Selenium is the 34th element and is incorporated into the 21st proteinogenic amino acid, selenocysteine (Sec). There are ~25 known genes in the human selenoproteome; however, the physiological roles, function and biochemical characterisation of several selenoproteins remains rudimentary. The single unifying factor of selenoproteins is that they all contain at least one Sec residue, which is inserted into nascent polypeptide chains in response to the UGA stop codon [1]. This phenomenon occurs in response to the Sec insertion sequence (SECIS) recoding UGA to the Sec codon [2]. Dietary selenium (Sec and selenomethionine) is phosphorylated by selenophosphate synthase 2 (SPS) and added to phosphoserine via selenocysteine synthetase (SecS), which produces Sec-tRNA^[Ser]Sec^ [2], allowing incorporation for Sec into proteins.

Levels of dietary selenium are known to have severe implications in cardiovascular health, endocrine system homeostasis, immune system regulation, musculoskeletal health and reproductive fertility [3,4]. The consequences of atypical dietary selenium intake are clear [5,6,7,8,9], although the implications of dietary selenium deficiency to the function of individual selenoproteins during pregnancy is poorly defined. Incorporation of Sec has been hypothesized to be the limiting step for selenoprotein biosynthesis and is primarily responsible for the regulation of selenoprotein expression by dietary selenium, with nonsense-mediated decay (NMD) [2]. Gene expression of stress-related selenoproteins, under conditions of selenium deficiency, are shown to decrease, which is likely due to NMD causing mRNA turnover [10]. This has been supported by ribosome profiling studies, where selenium availability affects gene expression of selenoproteins, causing depletion of glutathione peroxidase-1 (GPx-1) and several other “stress-related” selenoproteins [11]. This process is hypothesized to be responsible for the “hierarchy” of selenoprotein expression.

Within the human body, selenium concentration per gram of tissue is highest within the thyroid gland, with the highest amount of total selenium stored in the liver; however, it also accumulates within the brain, reproductive organs and other endocrine glands [12]. Selenium is regulated differently within each respective tissue—when an individual is selenium deplete, selenoprotein expression shows pronounced reduction in liver, kidneys and heart, whereas the brain retains selenium preferentially [12,13]. Although the role of selenium in human conception and pregnancy is extensively researched [14], the implications of selenium deficiency during pregnancy on the selenotranscriptome in maternal, foetal and offspring tissues remains unclear.

Previous studies have investigated the implications of aberrant selenium levels on mitochondrial function [3]. The thioredoxin system—comprised of thioredoxin, thioredoxin reductase (TrxR) and NADPH—is widely expressed, and has important antioxidant functions [15]. There are three forms of TrxR, all of which are selenoproteins: TrxR1 is cytosolic, TrxR2 is mitochondrial and TrxR3 is testis-specific [16]. The other seleno-dependent redox family are the glutathione peroxidases (GPx), which form part of the glutathione disulphide system and are responsible for the reduction of hydrogen peroxide to water [15]. There are several isoforms of GPx: GPx1 is the most abundantly and ubiquitously expressed, localised in the cytoplasm, and GPx3 is abundant in plasma, though in mice GPx3 has a role in organogenesis [16]. Selenium deficiency has been shown to alter antioxidant capacity and mitochondrial function due to alterations in TrxR and GPx systems, with suggestions of inducing pregnancy disorders such as preeclampsia, autoimmune thyroiditis and gestational diabetes mellitus [14,17].

Emerging evidence indicates that selenoproteins involved in endoplasmic reticulum (ER) homeostasis may have a fundamental role in regulating cellular and inflammatory stress responses [18]. Resident selenoproteins of the ER include: iodothyronine deiodinase 2 (DIO2), 15 kDa selenoprotein (*SelenoF*) and selenoproteins N, K, M, N, S and T [19]. There is developing evidence supporting the pivotal role that these selenoproteins have in maintaining ER stress responses, ER redox states and calcium signalling; however, characterisation of the physiological roles and biological function of these ER resident selenoproteins, as well as the TrxR and GPx systems, in pregnancy studies is sparse. 

Given the importance of selenium and selenoproteins in maintaining cellular function in response to physiological stressors such as pregnancy, delineating the effect of selenium deficiency during pregnancy on selenoprotein gene expression is necessary. This study aimed to characterise the effects of a maternal selenium deficiency on selenoprotein expression, across several tissues, in a pregnant rodent model. Furthermore, we explored whether selenoprotein expression in offspring was affected by exposure to selenium deficiency during development and defined possible implications. This animal model has been utilised in previous studies [20,21], with offspring undergoing additional procedures that are not hypothesized to have influenced selenoprotein expression.

## 2. Results

The effect of selenium deficiency on selenoprotein gene expression has previously been investigated; however, the effects of selenium deficiency concomitant with pregnancy has not previously been characterised. It is also important to monitor how this insult may affect multiple organ systems as well as placental, foetal and offspring gene expression. The effects of selenium deficiency on the expression of selenoproteins in maternal tissues is summarised in Table 1. Furthermore, the effects of selenium deficiency (treatment) on the placenta, foetal tissues and offspring tissues is summarised in Table 1, with differences in expression between sex summarised in Table 2.

### 2.1. Maternal Selenoprotein Expression

The expression levels of 14 selenoproteins were analysed in maternal liver, kidney and heart tissue at E18.5 (Table 3). Gene expression of *Txnrd1* was significantly reduced (*p* < 0.05) in the heart at E18.5; however, there was no changes in *Txnrd1* expression in the liver or kidneys. Expression of *Txnrd2* was also significantly reduced (*p* < 0.05) in the heart at E18.5, with no changes in liver or kidneys as well. *GPx1* and *GPx3* were reduced in all maternal tissues investigated. Most notably, *GPx1* was most significantly reduced (*p* < 0.01) in the heart, as was *GPx3* (*p* < 0.001).

Expression of iodothyronine deiodinases (DIOs)–selenoproteins important for thyroid hormone metabolism–were also determined in maternal tissues as shown in Table 3. Expression of *DIO1* was reduced (*p* < 0.01) in the liver of pregnant mice on a selenium deficient diet with no changes in *DIO1* expression within the kidneys. There were no changes in *DIO2* expression within either the kidney or the heart. Although examined, expression of *DIO1* in the heart, *DIO2* in the liver as well as *DIO3*, was undetermined.

Several selenoproteins that have not previously been implicated in human disease were also investigated in maternal tissues. *SelenoF* was reduced (*p* < 0.05) within the liver of pregnant mice on a selenium restricted diet, with no changes in the kidneys or heart. Expression of *SelenoM* was significantly reduced (*p* < 0.05) in all maternal tissues at E18.5. *SelenoT* expression in the heart was also reduced (*p* < 0.05) in pregnant mice on a low selenium diet, with no changes observed within the liver or kidney. Furthermore, expression of *SelenoK* was not altered in the tissues studied.

*SelenoN, SelenoP* and *SelenoS* are selenoproteins that have been previously implicated in disease. *SelenoN* was reduced (*p* < 0.01) within the kidneys of pregnant mice on a low selenium diet at E18.5, though no changes were observed in the heart. Furthermore, *SelenoP* was reduced (*p* < 0.05) in both the kidneys and heart, with preservation of expression in the liver. Expression of *SelenoS* was not different in either the kidneys or the heart; however, expression of *SelenoS* was significantly reduced (*p* < 0.05) in the liver of pregnant mice on a low selenium diet at E18.5. *SelenoN* was also examined in the liver; however, the expression levels were below the level of detection. 

### 2.2. Placental Selenoprotein Expression

Real-time qPCR data on placental expression of *Txnrd1* and *2, GPx1* and *3, DIO1* and *2* and *3, SelenoN* as well as *SelenoP* were analysed at embryonic Day 18.5 (Table 4). mRNA levels of both *DIO2 and DIO3* were reduced (*P*_trt_ < 0.05) with further post hoc analysis indicating the reduction observed in *DIO2* expression occurred predominantly in placentas of female foetuses (*p* < 0.05). Table 4 also illustrates expression of *SelenoF*, *SelenoK*, *SelenoS*, *SelenoM* and *SelenoT*, selenoproteins that have not previously been implicated in disease processes or pregnancy abnormalities. Maternal selenium deficiency did not have any significant impact on expression of any of these selenoproteins in the placenta at E18.5. Placental expression of selenoproteins that have been implicated in disease were also investigated. Gene expression of *SelenoN* (*P*_trt_ < 0.001) and *SelenoP* (*P*_trt_ < 0.05) were significantly reduced regardless of foetal sex.

### 2.3. Fetal Selenoprotein Expression

**Figure 1:**
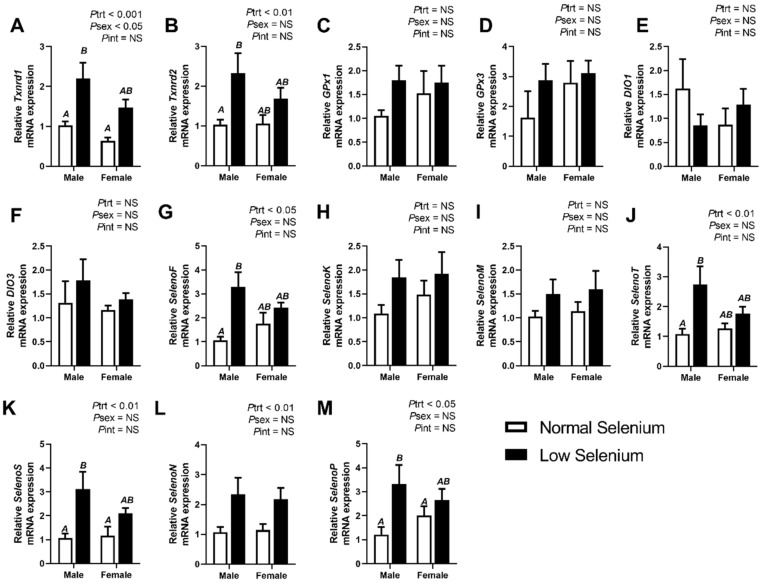
Effects of selenium deficiency on selenoprotein expression in fetal liver at E18.5. Expression in male and female fetal livers, at E18.5, of; (**A**) thioredoxin reductase 1, (**B**) thioredoxin reductase 2, (**C**) glutathione peroxidase 1, (**D**) glutathione peroxidase 3, (**E**) deiodinase 1, (**F**) deiodinase 3, (**G**) selenoprotein F, (**H**) selenoprotein K, (**I**) selenoprotein M, (**J**) selenoprotein T, (**K**) selenoprotein S. (**L**) selenoprotein N and (**M**) selenoprotein P. Data are mean ± SEM and analysed by two-way ANOVA with treatment (*P*_trt_) and sex (*P*_sex_) as major factors. *P*_int_ represents the interaction between treatment and sex. Significance was determined as *p* < 0.05. If a major effect of treatment, sex or an interaction between treatment and sex was identified, then a Sidak posthoc test was performed. Significant differences between groups as detected by posthoc analysis are denoted by different letters where “A” is different from “B”, but not “AB”. The groups are Normal male, *n* = 6 (6 animals from 6 separate litters); Low male, *n* = 6 (from 6 separate litters); Normal female, *n* = 6 (from 6 separate litters); Low female, *n* = 6 (from 6 separate litters).

**Figure 2:**
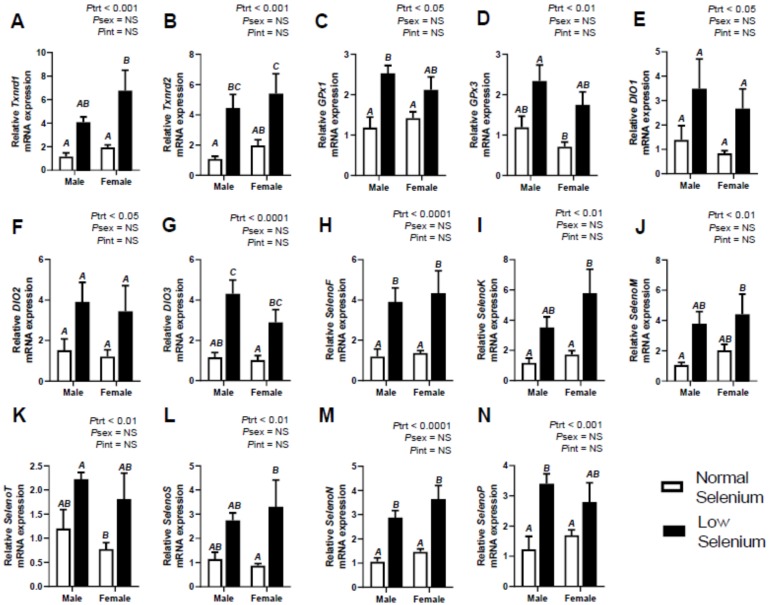
Effects of selenium deficiency on selenoprotein expression in fetal kidneys at E18.5. Expression in male and female fetal kidneys, at E18.5, of; (**A**) thioredoxin reductase 1, (**B**) thioredoxin reductase 2, (**C**) glutathione peroxidase 1, (**D**) glutathione peroxidase 3, (**E**) deiodinase 1, (**F**) deiodinase 2, (**G**) deiodinase 3, (**H**) selenoprotein F, (**I**) selenoprotein K, (**J**) selenoprotein M, (**K**) selenoprotein T, (**L**) selenoprotein S, (**M**) selenoprotein N and (**N**) selenoprotein P. Data are mean ± SEM and analysed by two-way ANOVA with treatment (*P*_trt_) and sex (*P*_sex_) as major factors. *P*_int_ represents the interaction between treatment and sex. Significance was determined as *p* < 0.05. If a major effect of treatments, sex or an interaction between treatment and sex was identified, then a Sidak posthoc test was performed. Significant differences between groups as detected by posthoc analysis are denoted by different letters where “A” is different from “B” which is different from “C”. “A” is not different from “A” or “AB”, “B” is not different from “B”, “AB” or “BC”. The groups are Normal male, *n* = 6 (6 animals from 6 separate litters); Low male, *n* = 6 (from 6 separate litters); Normal female, *n* = 6 (from 6 separate litters); Low female, *n* = 6 (from 6 separate litters).

**Figure 3:**
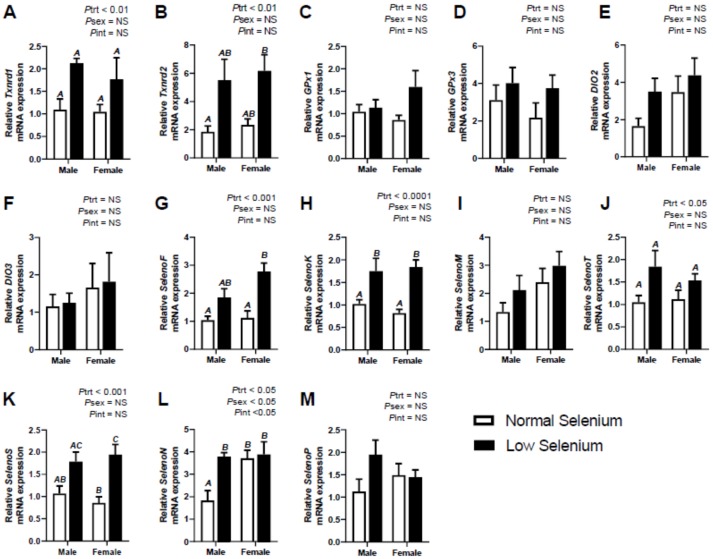
Effects of selenium deficiency on selenoprotein expression in fetal hearts at E18.5. Expression in male and female fetal hearts, at E18.5, of; (**A**) thioredoxin reductase 1, (**B**) thioredoxin reductase 2, (**C**) glutathione peroxidase 1, (**D**) glutathione peroxidase 3, (**E**) deiodinase 1, (**F**) deiodinase 3, (**G**) selenoprotein F, (**H**) selenoprotein K, (**I**) selenoprotein M, (**J**) selenoprotein T, (**K**) selenoprotein S, (**L**) selenoprotein N and (**M**) selenoprotein P. Data are mean ± SEM and analysed by two-way ANOVA with treatment (*P*_trt_) and sex (*P*_sex_) as major factors. *P*_int_ represents the interaction between treatment and sex. Significance was determined as *p* < 0.05. If a major effect of treatment, sex or an interaction between treatment and sex was identified, then a Sidak posthoc test was performed. Significant differences between groups as detected by posthoc analysis are denoted by different letters where “A” is different from “B” which is different from “C”. “A” is not different from “A”, “AB” or “AC”, while “B” is not different from “B”, or “AB”. The groups are Normal male, *n* = 6 (6 animals from 6 separate litters); Low male, *n* = 6 (from 6 separate litters); Normal female, *n* = 6 (from 6 separate litters); Low female, *n* = 6 (from 6 separate litters).

Selenoprotein expression was also investigated in foetal tissues at E18.5. Figure 1, Figure 2 and Figure 3 illustrate the expression of selenoproteins in liver, kidneys and heart of both male and female foetuses, respectively. At E18.5, expression of *Txnrd1* was increased (*P*_trt_ < 0.01) in all three tissues, more so in the liver and kidneys (*P*_trt_ < 0.001). Expression of *Txnrd1* was also lower (*P*_sex_ < 0.05) within the livers of female mice, irrespective of treatment (Figure 1A). Post hoc analysis further indicated *Txnrd1* mRNA expression within the liver was increased, to a greater extent in males (*p* < 0.05) and within the kidneys in females (*p* < 0.01–Figure 2A). Expression of *Txnrd2* in foetal tissues reflected the observations of *Txnrd1* expression, with significant increases (*P*_trt_ < 0.01) in *Txnrd2* expression across all foetal tissues, most notably within the kidneys (*P*_trt_ < 0.001–Figure 2B). Interestingly, post hoc analysis also showed *Txnrd2* expression was increased, within male livers (*p* < 0.05–Figure 1B) as well as female kidneys (*p* < 0.05–Figure 2B).

Expression of *GPx1* was increased (*p* < 0.001) within the kidneys (Figure 2C) of foetuses at E18.5, as was *GPx3* (*P*_trt_ < 0.01–Figure 2D). Post hoc analysis indicated kidneys from male foetuses from selenium deficient dams had significantly higher *GPx1* expression (*p* < 0.01) than male foetuses from normal dams. There were no changes observed in either *GPx1 or GPx3* expression in the livers and hearts of foetuses due to maternal selenium deficiency. 

All DIO mRNA expression (*DIO1, DIO2* and *DIO3*) was significantly increased (*P*_trt_ < 0.05) within the kidneys of foetal mice from selenium deficient dams at E18.5, most notably *DIO3* (*P*_trt_ < 0.0001–Figure 2G). Post hoc analysis indicated kidneys from male foetuses from selenium deficient dams had significantly higher *DIO3* expression (*p* < 0.01) than male foetuses from normal dams. There were no changes in foetal *DIO1* expression in the liver (Figure 1E), *DIO2* in the heart (Figure 3E) or *DIO3* in the liver and heart. *DIO1* was examined in the foetal hearts; however, expression was undetermined. Furthermore, *DIO2* was examined in the foetal livers and expression was also undetermined. This reflects results from investigations into *DIO* mRNA expression in maternal tissues shown in Table 2.

The mRNA expression of *SelenoF* was significantly increased within the liver (*P*_trt_ < 0.01–Figure 1G), kidneys (*P*_trt_ < 0.001–Figure 2H) and heart (*P*_trt_ < 0.001–Figure 3G) of foetuses from selenium deficient dams. Post hoc analysis indicated male foetuses from selenium deficient dams had significantly higher *SelenoF* expression in the liver (*p* < 0.01) and kidneys (*p* < 0.05) than fetuses from normal selenium dams. Furthermore, post hoc analysis also showed that female foetuses from selenium deficient dams had significantly higher *SelenoF* expression in the kidneys (*p* < 0.05) and heart (*p* < 0.01) than normal selenium females. Expression of *SelenoK* was significantly increased (*P*_trt_ < 0.01) in the kidneys (Figure 2I) and heart (Figure 3H) in foetal tissues at E18.5. Post hoc analysis demonstrated that females from low selenium dams had significantly higher expression within both kidneys (*p* < 0.05) and heart (*p* < 0.01) compared to females from normal selenium dams. This also occurred in the hearts of males from low selenium litters (*p* < 0.05). *SelenoM* expression was also increased (*P*_trt_ < 0.01–Figure 2J) in the kidneys of foetuses from low selenium dams; however, there were no changes in expression of *SelenoM* in foetal hearts or livers. *SelenoT* mRNA expression was significantly increased within the liver (*P*_trt_ < 0.01–Figure 1J), kidneys (*P*_trt_ < 0.01–Figure 1K) and heart (*P*_trt_ < 0.05–Figure 3J) of foetuses from low selenium dams. Post hoc analysis demonstrated that males from selenium deficient dams had significantly increased *SelenoT* expression (*p* < 0.05) within the liver at E18.5 compared to foetuses from normal selenium dams.

Expression of *SelenoS* (Figure 1K)*, SelenoN* (Figure 1L) and *SelenoP* (Figure 1M) was significantly increased (*P*_trt_ < 0.05) within livers of mice from selenium deficient dams. Post hoc analysis further demonstrated that *SelenoS* mRNA expression within the liver was increased (*p* < 0.05), to a greater extent, in males from selenium deficient dams. Kidney expression of *SelenS* (Figure 2L)*, SelenoN* (Figure 2M) and *SelenoP* (Figure 2N) was also increased (*P*_trt_ < 0.01) in foetuses from selenium deficient dams, especially *SelenoN* (*P*_trt_ < 0.0001). Post hoc analysis further demonstrated that *SelenoN* and *SelenoP* mRNA expression within kidneys was increased, to a greater extent, in males from selenium deficient dams (*p* < 0.01). Post hoc analysis also indicated that *SelenoN* and *SelenoS* mRNA expression within the kidneys was increased, to a greater extent, in females from selenium deficient dams (*p* < 0.05). Furthermore, *SelenoS* was also increased (*P*_trt_ < 0.001) within hearts (Figure 3K) of foetuses from selenium deficient dams. Post hoc analysis further indicated that *SelenoS* expression within the heart was increased (*p* < 0.01) in females from selenium deficient dams compared to females from normal selenium dams. Lastly, female *SelenoN* expression in the heart (Figure 3L) was higher (*P*_sex_ < 0.05), regardless of treatment. There was no effect of maternal selenium deficiency on foetal expression of *SelenoP* within the heart (Figure 3M).

### 2.4. Selenoprotein Expression in the Offspring

**Figure 4:**
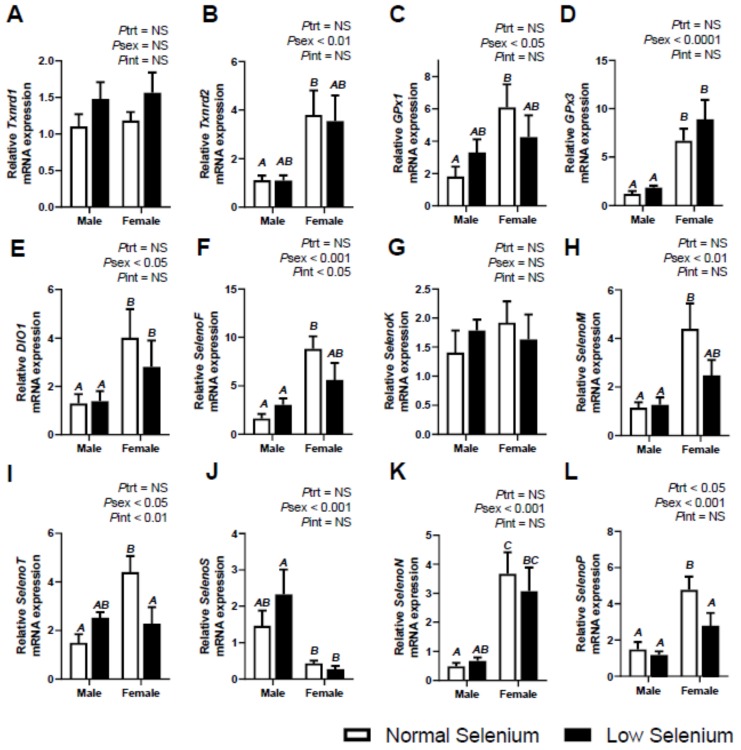
Effects of selenium deficiency on selenoprotein expression in offspring liver at PN180. Expression in male and female offspring livers, at PN180, of; (**A**) thioredoxin reductase 1, (**B**) thioredoxin reductase 2, (**C**) glutathione peroxidase 1, (**D**) glutathione peroxidase 3, (**E**) deiodinase 1, (**F**) selenoprotein F, (**G**) selenoprotein K, (**H**) selenoprotein M, (**I**) selenoprotein T, (**J**) selenoprotein S, (**K**) selenoprotein N and (**L**) selenoprotein P. Data are mean ± SEM and analysed by two-way ANOVA with treatment (*P*_trt_) and sex (*P*_sex_) as major factors. *P*_int_ represents the interaction between treatment and sex. Significance was determined as *p* < 0.05. If a major effect of treatment, sex or an interaction between treatment and sex was identified, then a Sidak posthoc test was performed. Significant differences between groups as detected by posthoc analysis are denoted by different letters where “A” is different from “B” which is different from “C”. “A” is not different from “A” or “AB”, while “B” is not different from “B”, “AB” or “BC”. The groups are Normal male, *n* = 8 (8 animals from 8 separate litters); Low male, *n* = 8 (from 8 separate litters); Normal female, *n* = 8 (from 8 separate litters); Low female, *n* = 6 (from 6 separate litters).

**Figure 5:**
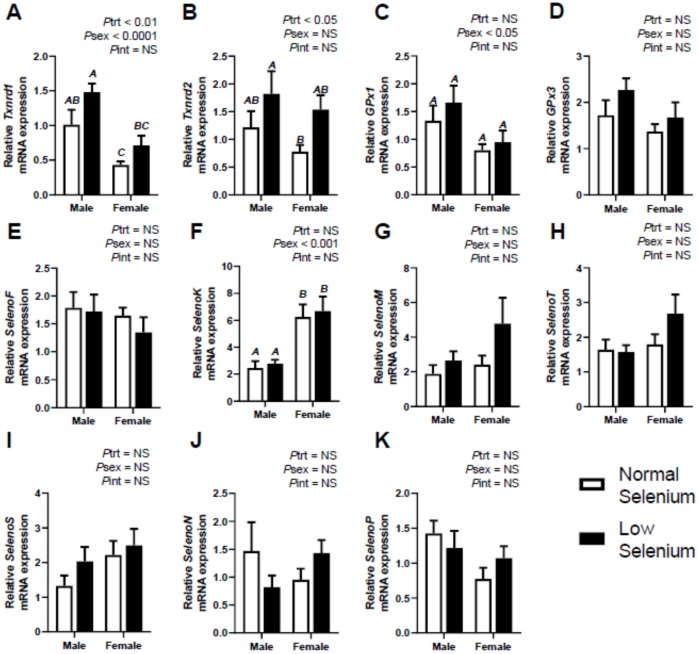
Effects of selenium deficiency on selenoprotein expression in offspring kidneys at PN180. Expression in male and female offspring kidneys, at PN180, of; (**A**) thioredoxin reductase 1, (**B**) thioredoxin reductase 2, (**C**) glutathione peroxidase 1, (**D**) glutathione peroxidase 3, (**E**) selenoprotein F, (**F**) selenoprotein K, (**G**) selenoprotein M, (**H**) selenoprotein T, (**I**) selenoprotein S, (**J**) selenoprotein N and (**K**) selenoprotein P. Data are mean ± SEM and analysed by two-way ANOVA with treatment (*P*_trt_) and sex (*P*_sex_) as major factors. *P*_int_ represents the interaction between treatment and sex. Significance was determined as *p* < 0.05. If a major effect of treatment, sex or an interaction between treatment and sex was identified, then a Sidak posthoc test was performed. Significant differences between groups as detected by posthoc analysis are denoted by different letters where “A” is different from “B” which is different from “C”. “A” is not different from “A” or “AB”, while “B” is not different from “B”, “AB” or “BC”. The groups are Normal male, *n* = 8 (8 animals from 8 separate litters); Low male, *n* = 8 (from 8 separate litters); Normal female, *n* = 8 (from 8 separate litters); Low female, *n* = 6 (from 6 separate litters).

**Figure 6:**
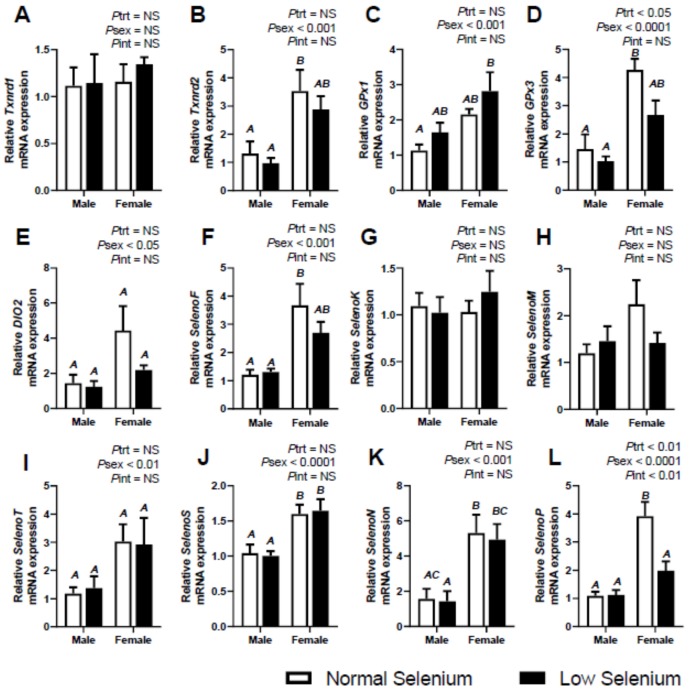
Effects of selenium deficiency on selenoprotein expression in offspring hearts at PN180. Expression in male and female offspring hearts, at PN180, of; (**A**) thioredoxin reductase 1, (**B**) thioredoxin reductase 2, (**C**) glutathione peroxidase 1, (**D**) glutathione peroxidase 3, (**E**) deiodinase 2, (**F**) selenoprotein F, (**G**) selenoprotein K, (**H**) selenoprotein M, (**I**) selenoprotein T, (**J**) selenoprotein S, (**K**) selenoprotein N and (**L**) selenoprotein P. Data are mean ± SEM and analysed by two-way ANOVA with treatment (*P*_trt_) and sex (*P*_sex_) as major factors. *P*_int_ represents the interaction between treatment and sex. Significance was determined as *p* < 0.05. If a major effect of treatment, sex or an interaction between treatment and sex was identified, then a Sidak posthoc test was performed. Significant differences between groups as detected by posthoc analysis are denoted by different letters where “A” is different from “B” which is different from “C”. “A” is not different from “A” or “AB”, while “B” is not different from “B”, “AB” or “BC”. The groups are Normal male, *n* = 8 (8 animals from 8 separate litters); Low male, *n* = 8 (from 8 separate litters); Normal female, *n* = 8 (from 8 separate litters); Low female, *n* = 6 (from 6 separate litters).

The effects of selenium deficiency during pregnancy on selenoprotein expression in male and female offspring at six months of age was also investigated (Figure 4, Figure 5 and Figure 6). The mRNA expression of *Txnrd1* was significantly increased (*P*_trt_ < 0.01) in the kidneys (Figure 5A) of offspring from selenium deficient litters irrespective of sex, as was expression of *Txnrd2* (*P*_trt_ < 0.05–Figure 5B). Expression of *Txnrd1* was also lower in female kidneys compared to males (*P*_sex_ < 0.01), regardless of treatment. Expression of *Txnrd2* was higher within female livers (*P*_sex_ < 0.01, Figure 4B) and female hearts (*P*_sex_ < 0.001–Figure 6B), irrespective of treatment. Maternal selenium deficiency was not associated with any changes in mRNA expression of *Txnrd1* or *Txnrd2* in the liver or heart.

Expression of glutathione peroxidases was also investigated in tissues at PN180. There was no effect of maternal selenium deficiency on offspring mRNA expression of *GPx1* in any tissue; however, there were differences in mRNA expression between sexes in all tissues. *GPx1* expression was higher in female livers (*P*_sex_ < 0.05–Figure 4C) and hearts (*P*_sex_ < 0.001–Figure 6C), though lower in kidneys (*P*_sex_ < 0.05–Figure 5C). Expression of *GPx3* was significantly lower (*P*_trt_ < 0.05) within the hearts (Figure 6D) of offspring from selenium deficient litters, though there were no changes in the liver or kidneys due to treatment. The expression of *GPx3* was also significantly higher (*P*_sex_ < 0.0001) within female livers and hearts, irrespective of treatment. 

Investigation into the expression of *DIOs* in PN180 offspring tissues was also conducted. Expression of *DIO1* was higher within female offspring livers (*P*_sex_ < 0.05–Figure 4E) when compared to males, irrespective of treatment. The mRNA expression of *DIO2* in male kidneys at PN180 was undetermined, although expression was detected in female kidneys (Normal selenium female 1.52 ± 0.42; Low selenium female 2.26 ± 0.48) as well as maternal kidneys (Table 2). Again, expression of *DIO1* in offspring heart and *DIO2* in the liver was investigated, though expression was undetermined, reflecting the results depicted in maternal tissues (Table 1) and foetal tissues (Figure 1, Figure 2 and Figure 3). Furthermore, *DIO3* expression was examined and was also undetermined, as was shown in maternal tissues (refer to Table 1). 

There was no effect of maternal selenium deficiency on PN180 mRNA expression of selenoproteins *F, K, M* and *T;* however, several sex differences were observed. *SelenoF* expression was higher (*P*_sex_ < 0.001) in female livers (Figure 4F) and hearts (Figure 6F) compared to males at PN180, regardless of treatment. Expression of *SelenoK* was also significantly higher (*P*_sex_ < 0.001–Figure 5F) within female kidneys at PN180. As well as *SelenoF,* expression of *SelenoM* (Figure 4H) and *SelenoT* (Figure 4I) was significantly higher (*P*_sex_ < 0.05) within the liver of females at PN180. Furthermore, the mRNA expression of *SelenoT* was higher (*P*_sex_ < 0.01–Figure 6I) in female hearts when compared to males, at PN180. An interaction between treatment and sex was also determined in *SelenoF* expression at PN180 (*P*_int_ < 0.05) within offspring livers as was *SelenoT* expression (*P*_int_ < 0.01).

Maternal selenium deficiency led to a reduction (*P*_trt_ < 0.05) in *SelenoP* expression within offspring livers (Figure 4L). A treatment effect was also observed in *SelenoP* expression within the hearts (*P*_trt_ < 0.01–Figure 6L) of offspring from selenium deficient litters. Sex differences in mRNA expression of *SelenoN, SelenoP* and *SelenoS* were observed in the livers and hearts at PN180 (*P*_sex_ < 0.001). All expression observed was higher in females at PN180 excluding *SelenoS* expression within the liver, which was lower in females (*P*_sex_ < 0.001). A significant interaction between treatment and sex was observed in *SelenoP* expression (*P*_int_ < 0.01) in offspring hearts at PN180. There were no effects of maternal selenium deficiency on selenoprotein expression of *SelenoN or SelenoS* in any tissue at PN180. There were also no sex differences in expression of *SelenoN, SelenoP* or *SelenoS* within the kidneys at PN180. 

## 3. Discussion

This study comprehensively profiled mRNA levels of 14 different selenoproteins in maternal, fetal and offspring tissue in a mouse model of selenium deficiency during pregnancy. We have demonstrated that dietary selenium deficiency alters mRNA expression in the placenta as well as the liver, kidneys and heart of pregnant mice, foetuses and offspring. Previously, we established that deficiency suppressed gestational weight gain, altered placental function, reduced foetal growth, and programmed metabolic disease in offspring [20]. Furthermore, thyroid and metabolic dysfunction were consistent at all stages investigated in the model [21]. In the present study we reiterate that it is a necessity to maintain dietary selenium intake during pregnancy to maintain selenoprotein transcription and that the liver, kidneys and heart all susceptible to selenium deficiencies. This study indicates that mild selenium deficiency, equivocal to that observed in human populations, is enough to cause significant transcriptional changes to selenoprotein expression. 

### 3.1. Maternal Selenoproteins

As selenium distribution throughout the body is heterogenous, dynamic and dependent on the availability of selenium, discovering that the expression of selenoproteins was differentially impacted in different tissues in this model was expected [22,23]. The mRNA expression of several selenoproteins were supressed in maternal tissues. Most notably, *GPx1*, *GPx3*, and *SelenoM* were universally reduced in all three tissues investigated: liver, kidneys and heart. Glutathione peroxidases are expressed ubiquitously throughout the body, found in virtually all cells and well renowned for responding very rapidly to alterations in selenium status [24]. In the “hierarchy of selenoproteins”, *GPx1* ranks the lowest, attributed to the instability of *GPx1* mRNA [25]; however additional selenium-responsive factors allow for stabilization of mRNA expression, explaining why gene expression preservation is different between tissues, as seen with the interaction of eukaryotic initiation factor 4a3 (eIF4a3) with selenoprotein mRNA, preventing SECIS binding protein 2 (SBP2) binding, and thus translation [26]. Consequently, the reduced expression observed in maternal tissues was expected [27]. Supressed *SelenoM* expression is associated with weight gain, due to exacerbated ER stress, reducing leptin sensitivity causing an increase in caloric intake and weight gain [28]. Although, gestational weight gain decreased in mice on a selenium deficient diet with no change in caloric intake [20], suggesting *SelenoM* expression in the brain may have been preserved. 

Within maternal livers, the expression of *DIO1* was also supressed, concurrent with reductions in *DIO2* and *DIO3* expression within the placenta. Patients with genetic SBP2 mutations, either homozygous or compound heterozygous, present with abnormal thyroid profiles exemplified with reduced triiodothyronine, high thyroxine with normal thyroid stimulating hormone levels [29]. This same mutation was associated with impaired synthesis of several selenoproteins including *GPx, Txnrd, SelenoN and SelenoP* [30]. Alteration in genotypic expression of several selenoproteins results in a variety of atypical phenotypes including growth retardation, myopathy and enhanced insulin sensitivity [31]; however, the lack in correlation between genotype and phenotype and complexity in the variability of affinity of SBP2 and SECIS element in genes makes it difficult to ascertain hierarchical reduction of translation efficiencies of different selenoproteins in the different tissues [30]. Although from evidence in models of selenium deficiency and selenoprotein knockout models it is still possible to infer possible outcomes of changes in selenoprotein expression. This study further supports that *DIO3* is only expressed in developing and generating tissues, including the placenta and foetal tissues, playing a critical role in foetal thyroid hormone concentrations and regulations of growth.

A novel finding in this study is that expression of *Txnrd1* and *Txnrd2* in the maternal heart was reduced. In rat hearts, selenium deficiency has also been associated with decreased expression in both *TrxR*, as well as *GPx1*, impairing ischemia-reperfusion recovery [32]. Kiermayer et. al. showed, in a knockout model of *Txnrd2*, myocardium contractile and metabolic function was induced, suggesting *Txnrd2* may be a modifier of heart failure during aging [33]. Conversely, at E18.5, expression of both *Txnrd1* and *Txnrd2* was significantly increased in all tissues. 

Expression of *SelenoP* was also supressed in the kidneys and heart of mothers, as well as the placenta. As *SelenoP* is a secreted glycoprotein and contains most of the selenium in plasma, it is hypothesized that selenium transport is controlled by *SelenoP* [34]. Synthesis of *SelenoP* has been proven to decrease concomitant to selenium deficiency, causing plasma concentrations to decline [35], although *SelenoP* expression has previously been shown to increase prior to pregnancy and reach maximum levels at full-term in mice [36].

### 3.2. Fetal Selenoproteins

As selenoprotein expression was reduced in all maternal tissues, changes in placental and foetal selenoprotein expression were expected; although, the increases in expression of selenoproteins of foetuses exposed to selenium deficiency in utero was not anticipated. Interestingly, all selenoproteins investigated had increased gene expression within the kidney. Additionally, *Txnrd1, Txnrd2, SelenoF, SelenoS, SelenoT, SelenoN* and *SelenoP* were all increased within the foetal liver and *Txnrd1*, *Txnrd*2, *SelenFf*, *SelenoS, SelenoK* and *SelenoT* were all increased in foetal hearts from selenium deficient dams. 

Currently, two known maternal-foetal selenium transfer mechanism are known, requiring *SelenoP* and apolipoprotein E receptor-2 (apoER2), to maintain selenium transfer to the foetus under selenium deplete circumstances [37]; however, in our model, selenium levels were depleted in maternal, placental and foetal tissues, indicating the reduced maternal *SelenoP* expression observed was enough to alter distribution of selenium [38]. Other mechanisms may be interacting to cause and increase in selenoprotein expression in foetal tissues. Sec lyase (SCL) is an enzyme that degrades Sec residues to form L-alanine and selenium, increasing selenium bioavailability [39]. SCL can interact with both SPS1 and SPS2, which promote de novo synthesis of selenophosphate and Sec recycling through salvaging of selenium respectively [39]. Upregulation of SCL in maternal tissues and upregulation of SPS1/2 in foetal tissues would encourage an increase in Sec-tRNA^[Ser]Sec^ [2]. An increase in Sec-tRNA^[Ser]Sec^ in foetal tissues would permit Sec incorporation into proteins, preventing NMD and allowing biosynthesis of selenoproteins. Upregulation of SCL would also explain the significant reductions in selenoprotein expression within tissues of the mothers, although these factors were not investigated.

In mice, selenium deficiency has been previously demonstrated to increase expression of *GPx2* in the gastrointestinal tract [40]; however, this is the first study to investigate the effects of dietary selenium deficiency on foetal selenoprotein expression. Expression of *DIO2* and *SelenoN* in kidneys has also been recognised to upregulate in response to dietary selenium deficiency [41], with age also playing a factor in upregulation of several others. Cao et. al. also observed increased selenoprotein expression in response to dietary selenium deficiency, although they did not propose a mechanism to explain this finding [41], Transcriptional regulation of selenoproteins is multifaceted and complex, with overexpression of selenoproteins primarily associated with combatting oxidative stress and immunocompromise [42].

As processivity of Sec residues is intrinsic and efficiency is reliant on selenium levels [43], it is tempting to speculate that foetal selenoprotein expression is maintained during in utero exposure to selenium deficiency by upregulation of SECIS and SBP2 elements, at the expense to the mother and placenta [44]. This way, even in the presence of a selenium deficit, the foetus could maintain normal development. Here, we provide insights into foetal expression of selenoproteins in normal and selenium deplete pregnancies; we indicate, through unknown mechanisms, that selenoprotein expression within foetal tissues is increased when exposed to a selenium deficit in utero.

### 3.3. Offspring Selenoproteins

At PN180, a strong sexual dimorphic genotypic expression of selenoproteins was observed. Several studies have previously demonstrated sex-specific expression of selenoproteins [24]. Evidently, when selenium deplete rats are given an injection of selenium, females are able to retain the micronutrient more efficiently than males in most organs other than reproductive organs [45]. This may account for the increase in selenoprotein expression in several female organs at PN180, when compared to males. Sex-specific differences in selenoprotein expression is generally attributed to differing circulating steroid hormone levels linked to autonomous steroid hormone-independent cellular pathways in selenoprotein transcription/translation processes [46,47]. In comparison, sexual dimorphism by dietary selenium deficiency was less remarkable at E18.5

Treatment effects were noted at PN180, including an increase in *Txnrd*1 and 2 expression in the kidney, as well as reduced *GPx3* expression in the heart and reduced *SelenoP* in both liver and heart in mice from selenium deficient litters. In human, expression of thioredoxin systems generally increases in response to inflammation associated with hypertension and atherosclerotic plaques [48,49]. Furthermore, reduced *GPx3* expression is a contributing factor to circulatory diseases, and a contributing factor to developing kidney-induced cardiac disease [50]. Expression of *SelenoP* is regulated by insulin and glucocorticoid levels, with reduced hepatic *SelenoP* associated with irregular distribution of selenium throughout the body [51]. Together, these observed treatment effects at PN180 indicate programming changes due to in utero and perinatal selenium deficiency, which may contribute to circulatory diseases. Given these findings, understanding and delineating the effects of selenium deficiency during foetal development on foetal and offspring selenoproteome gene expression, as well as protein expression and activity, is pertinent. Future studies should aim at further delineating the relationship between dietary selenium and genomic expression of selenoproteins, specifically during foetal development.

## 4. Materials and Methods

### 4.1. Animal Model

All experiments were approved by Griffith University Animal Ethics Committee (Ethics no. MSC/01/16/AEC, 11^th^ of April 2016) and were conducted in accordance with the Australian Code of Practice for Care and Use of Animals for Scientific purposes. Experimental design as well as housing and husbandry of animals was in accordance with the “Animals in Research: Reporting In Vivo Experiments” (ARRIVE) guidelines for DOHaD research [52].

Briefly, female C57BL/6 mice were obtained from the Animal Resources Centre (ARC, Perth, Western Australia). After one-week acclimatisation, mice were placed on a four-week pre-pregnancy dietary exposure of a normal selenium (NS; *n* = 16) or low selenium (LS; *n* = 16) chow. Subsequent to this, half the female mice from both dietary groups were either culled at E18.5 via cervical dislocation (NS, *n* = 8; LS, *n* = 8) or permitted to litter down (NS, *n* = 8; LS, *n* = 8) for offspring studies. 

At E18.5, maternal liver, kidneys and heart were collected, wet weighed and then snap frozen, followed by storage at −80 °C. Individual placentas and foetuses were collected from the uterine horn and foetal liver, kidneys and heart were also collected, snap frozen and stored at −80 °C.

Offspring were monitored daily from postnatal day 8 (PN8) and weaned at PN24. After weaning, offspring were group housed by litter and sex, and were placed on normal diets and standard animal housing conditions as previously described [21]. At PN180, offspring were culled via cervical dislocation, followed by collection of liver, kidneys and heart which were wet weighed, snap frozen and stored at −80 °C.

### 4.2. Fetal Genotyping

Foetal tails were collected; DNA was isolated and purified from this tissue using the DNeasy Blood & Tissues Kit (Qiagen, Melbourne, Australia; Cat. no. 69506). The sex-determining region Y (*Sry*, NM_011564) was used to determine sex with Taqman Gene expression technology. Assessment of the samples was conducted in duplicate on a StepOnePlus Real-Time PCR System (Applied Biosystems, Foster City, California, USA) as previously described. 

### 4.3. Quantitative PCR

RNA extraction, conversion of RNA into cDNA, and qPCR analysis utilized in this study have all been previously described [20]. Briefly, RNA was extracted using the RNAeasy mini kit (Qiagen, Melbourne, Australia; Cat. no. 74106). The heart tissue went through an additional step with addition of proteinase K (20 mg/mL) and incubation at 55 °C after homogenisation to remove connective tissue. RNA concentration was determined using absorbance spectrophotometry and RNA was converted into cDNA for qPCR analysis using the Bio-Rad iScript gDNA clear cDNA synthesis kit (Hercules, California, USA; Cat. no. 172-5035). Approximately 20 ng of cDNA in 10 µL per reaction was used to perform qPCR, with samples run in duplicate. All PCRs were performed using the same thermocycling parameters using a StepOne Real-Time PCR system (Applied Biosystems, Foster City, California, USA). Initial activation step at 95 °C for 2 min, followed by 40 cycles of denaturation at 95 °C for 5 s and combined annealing/extension 60 °C for 10 s. All PCR reactions were performed in accordance with the *MIQE* guidelines.

Measurements of mRNA expression of selenoproteins was performed using KiCqStart SYBR green PCR primers (Sigma-Aldrich, Missouri, USA) as described in Table 5. No product was detected in the non-template control on any run, and a melt curve analysis demonstrated a single spike for all genes measured. Several potential housekeeper genes were investigated and assessed based on previous research; however, the reference genes utilized for each tissue where specifically chosen as they demonstrated consistent expression in the respective tissue and were not impacted by neither treatment nor sex. After assessment of several potential reference genes, the housekeepers as described in Table 5 were used. Briefly, maternal livers were normalised to the geometric mean of *Actb, Ubc* and *Top1*, with kidneys normalised the geometric mean of *Actb* and *Ubc* and hearts normalised to the geometric mean of *Actb* and *Hprt*. Placentas were normalised to the geometric mean of *Actb* and *Ubc.* At E18.5, mRNA expression was normalised to the geometric mean of *Gapdh* and *Rn18s* for livers and kidneys and to the geometric mean of *Gapdh* and *Actb* for the heart. At PN180, livers were normalised to the geometric mean of *Actb* and *Top1*, kidneys to *Actb* and *Ubc* and hearts to *Actb* and *Hprt*. Final expression for all selenoproteins investigated was calculated using the 2^−ΔΔ*C*t^ method with data expressed as fold change relative to the expression of housekeeping genes. 

### 4.4. Statistical Analysis

qPCR data for selenoprotein expression within maternal tissues was statistically analysed using an Unpaired *t*-test. Placental, E18.5 and PN180 tissues were all analysed using two-way ANOVA (GraphPad Prism 8.2.1) with the main effects of maternal selenium deficiency (Treatment; *P*trt) and sex (Sex; *P*sex) with any interactions between treatment and sex (Interaction; *P*int) assessed. When a major effect of treatment, sex or an interaction between treatment and sex was detected, Sidak post hoc analysis was performed. All data is presented as mean ± SEM with *P* < 0.05 considered statistically significant. Differences between groups are denoted by different letters where “A” is different from “B”, but not “AB”. The offspring groups were analysed by selecting 1 male pup and 1 female pup from each of the treated or non-treated mothers (*n* = 6–8 litters of each treatment). 

## 5. Conclusions

This study comprehensively investigated the effects of maternal selenium deficiency on the gene expression of several selenoproteins in multiple tissues. We have demonstrated that selenium deficiency caused a reduction of several maternal selenoproteins in the liver, kidneys, heart and placenta; however, our results strongly suggest that foetuses adapt and increase expression. This study is the first to demonstrate that maternal selenium deficiency causes this increase in selenoprotein expression in both male and female foetal tissues, with mechanisms remaining elusive. Furthermore, this study reiterates that the effects of selenium are sexually dimorphic, with the exposure to a selenium deficit in utero and perinatally causing sexual differences in offspring selenoprotein expression. These results emphasise the importance of dietary selenium in maintaining the selenotranscriptome, and that deficiency of selenium during pregnancy has genomic programming potential.

## Figures and Tables

**Table 1:** Summary table of the effects of selenium deficiency on selenoprotein expression in various maternal and offspring tissues.

Treatment										
Gene	Maternal			Placenta	E18.5			PN180		
	Liver	Kidney	Heart		Liver	Kidney	Heart	Liver	Kidney	Heart
*Txnrd1*	-	-	↓	-	↑	↑	↑	-	↑	-
*Txnrd2*	-	-	↓	-	↑	↑	↑	-	↑	-
*GPx1*	↓	↓	↓	-	-	↑	-	-	-	-
*GPx3*	↓	↓	↓	-	-	↑	-	-	-	↓
*DIO1*	↓	-	N/A	-	-	↑	N/A	-	N/A	N/A
*DIO2*	N/A	-	-	↓	N/A	↑	-	N/A	N/A	-
*DIO3*	N/A	N/A	N/A	↓	-	↑	-	N/A	N/A	N/A
*SelenoF*	↓	-	-	-	↑	↑	↑	-	-	-
*SelenoS*	↓	-	-	-	↑	↑	↑	-	-	-
*SelenoK*	-	-	-	-	-	↑	↑	-	-	-
*SelenoM*	↓	↓	↓	-	-	↑	-	-	-	-
*SelenoT*	-	-	↓	-	↑	↑	↑	-	-	-
*SelenoN*	N/A	↓	-	↓	↑	↑	-	-	-	-
*SelenoP*	-	↓	↓	↓	↑	↑	-	↓	-	↓

- indicates no significant difference. ↑ indicates significantly increased expression in the low selenium group. ↓ indicates significantly decreased expression in the low selenium group. N/A indicates expression was undetermined and therefore statistical analysis was not applicable.

**Table 2:** Summary table of the effects of sex on selenoprotein expression in various foetal and offspring tissues.

Sex							
Gene	Placenta	E18.5			PN180		
		Liver	Kidney	Heart	Liver	Kidney	Heart
*Txnrd1*	-	↓	-	-	-	↓	-
*Txnrd2*	-	-	-	-	↑	-	↑
*GPx1*	-	-	-	-	↑	↓	↑
*GPx3*	-	-	-	-	↑	-	↑
*DIO1*	-	-	-	N/A	↑	N/A	N/A
*DIO2*	-	N/A	-	-	N/A	N/A	↑
*DIO3*	-	-	-	-	N/A	N/A	N/A
*SelenoF*	-	-	-	-	↑	-	↑
*SelenoS*	-	-	-	-	↓	-	↑
*SelenoK*	-	-	-	-	-	↑	-
*SelenoM*	-	-	-	-	↑	-	-
*SelenoT*	-	-	-	-	↑	-	↑
*SelenoN*	-	-	-	↑	↑	-	-
*SelenoP*	-	-	-	-	↑	-	↑

- indicates no significant difference. ↑ indicates significantly increased expression in female mice, irrespective of treatment. ↓ indicates significantly decreased expression in female mice, irrespective of treatment. N/A indicates expression was undetermined and therefore statistical analysis was not applicable.

**Table 3:** Effects of selenium deficiency on selenoprotein expression in various maternal tissues.

Maternal									
	Liver			Kidney			Heart		
Gene	Normal	Low	P	Normal	Low	P	Normal	Low	P
*Txnrd1*	1.01 ± 0.06	1.16 ± 0.23	NS	1.05 ± 0.12	1.21 ± 0.18	NS	1.17 ± 0.24	0.63 ± 0.06	**0.0497**
*Txnrd2*	1.05 ± 0.13	0.91 ± 0.32	NS	1.21 ± 0.27	1.31 ± 0.45	NS	1.22 ± 0.28	0.56 ± 0.10	**0.0477**
*Gpx1*	1.00 ± 0.17	0.44 ± 0.15	**0.0271**	1.24 ± 0.14	0.79 ± 0.13	**0.0369**	1.03 ± 0.10	0.51 ± 0.12	**0.0051**
*Gpx3*	1.12 ± 0.18	0.60 ± 0.16	**0.0473**	1.12 ± 0.21	0.56 ± 0.11	**0.0393**	1.23 ± 0.30	0.34 ± 0.05	**0.0088**
*DIO1*	1.08 ± 0.16	0.54 ± 0.19	**0.0458**	1.26 ± 0.27	1.64 ± 0.42	NS	-	-	-
*DIO2*	-	-	-	1.11 ± 0.16	0.95 ± 0.16	NS	1.19 ± 0.26	1.17 ± 0.29	NS
*DIO3*	-	-	-	-	-	-	-	-	-
*SelenoF*	1.18 ± 0.21	0.64 ± 0.10	**0.0463**	1.05 ± 0.12	1.01 ± 0.14	NS	1.03 ± 0.11	0.78 ± 0.17	NS
*SelenoS*	1.18 ± 0.20	0.66 ± 0.08	**0.0370**	1.06 ± 0.12	1.17 ± 0.17	NS	1.03 ± 0.12	0.87 ± 0.16	NS
*SelenoK*	1.06 ± 0.14	0.73 ± 0.20	NS	1.03 ± 0.10	1.08 ± 0.08	NS	1.03 ± 0.11	0.70 ± 0.11	NS
*SelenoM*	1.01 ± 0.06	0.61 ± 0.18	**0.0353**	1.02 ± 0.08	0.68 ± 0.10	**0.0169**	1.11 ± 0.20	0.39 ± 0.10	**0.0157**
*SelenoT*	1.09 ± 0.18	0.66 ± 0.13	NS	1.03 ± 0.08	0.89 ± 0.10	NS	1.08 ± 0.17	0.61 ± 0.11	**0.0297**
*SelenoN*	-	-	-	1.01 ± 0.05	0.64 ± 0.10	**0.0055**	1.36 ± 0.38	0.76 ± 0.12	NS
*SelenoP*	1.05 ± 0.11	0.78 ± 0.21	NS	1.05 ± 0.15	0.63 ± 0.05	**0.0183**	1.05 ± 0.14	0.59 ± 0.08	**0.0130**

Maternal selenoprotein gene expression in the liver, kidney and heart. Data are mean ± SEM and analysed by Unpaired *t*-tests. All values are relative mRNA expression of specified genes based on the 2^−ΔΔ*C*t^ method. Bold text indicates significance, where *p* < 0.05. *n* = 8. NS; non-significant.

**Table 4:** Effects of selenium deficiency on selenoprotein expression in the placenta at E18.5.

E18.5 Placenta							
Gene	Male		Female		*P*_trt_	*P*_sex_	*P*_int_
	Normal	Low	Normal	Low			
*Txnrd1*	1.27 ± 0.13	1.33 ± 0.12	1.15 ± 0.18	1.47 ± 0.23	NS	NS	NS
*Txnrd2*	1.38 ± 0.18	2.12 ± 0.29	1.37 ± 0.48	1.62 ± 0.28	NS	NS	NS
*Gpx1*	1.12 ± 0.03	1.07 ± 0.05	1.00 ± 0.18	1.12 ± 0.04	NS	NS	NS
*Gpx3*	1.71 ± 0.45	1.33 ± 0.47	1.46 ± 0.44	1.64 ± 0.24	NS	NS	NS
*DIO1*	0.85 ± 0.28	2.55 ± 0.36	0.88 ± 0.18	3.53 ± 0.95	NS	NS	NS
*DIO2*	2.13 ± 0.91	1.39 ± 0.52	3.10 ± 1.00	1.08 ± 0.23 ^a^	**0.0422**	NS	NS
*DIO3*	1.03 ± 0.42	0.77 ± 0.20	0.76 ± 0.18	0.58 ± 0.13	**0.0491**	NS	NS
*SelenoF*	0.91 ± 0.07	1.39 ± 0.19	1.29 ± 0.29	1.50 ± 0.12	NS	NS	NS
*SelenoS*	0.95 ± 0.12	1.21 ± 0.17	1.13 ± 0.24	1.11 ± 0.04	NS	NS	NS
*SelenoK*	1.02 ± 0.04	1.09 ± 0.06	1.23 ± 0.21	1.68 ± 0.25	NS	NS	NS
*SelenoM*	0.96 ± 0.11	1.32 ± 0.12	1.53 ± 0.26	1.46 ± 0.13	NS	NS	NS
*SelenoT*	0.91 ± 0.11	0.99 ± 0.11	0.96 ± 0.10	1.19 ± 0.10	NS	NS	NS
*SelenoN*	0.90 ± 0.12	0.62 ± 0.11	1.03 ± 0.06	0.60 ± 0.03 ^c^	**0.0002**	NS	NS
*SelenoP*	1.11 ± 0.29	0.66 ± 0.28	0.64 ± 0.22	0.72 ± 0.14	**0.0491**	NS	NS

Placental selenoprotein gene expression at E18.5. Data are mean ± SEM. All values are relative mRNA expression of specified genes based on the 2^−ΔΔ*C*t^ method. Analysis is by two-way ANOVA with treatment (*P*_trt_) and sex (*P*_sex_) as major factors. *P*_int_ represents the interaction between trt and sex. Bold text indicates significance, where *p* < 0.05. Significance between treatment groups of the same sex designated by Sidak post hoc testing is indicated by *a* = *p* < 0.05, *b* = *p* < 0.01 and *c* = *p* < 0.001. Normal male, *n* = 8 (8 male placentas from 8 separate litters); Low male, *n* = 8 (across 8 litters); Normal female, *n* = 8 (across 8 litters); Low female, *n* = 8 (across 8 litters). NS; non-significant.

**Table 5:** qPCR primer list for 14 selenoproteins and 5 housekeeping genes.

Group	Gene Name	*Gene Acronym*	Accession Number	Primer Sequence
Selenoproteins	Thioredoxin Reductase 1	*Txnrd1*	NM_001042513	F’ TCCCAACGAAAATTGAACAGR’ TGTTAAATTCGCCCTCTATG
	Thioredoxin Reductase 2	*Txnrd2*	NM_013711	F’ GAATCACAAGTGACGACATCR’ AAAGATGACATTTGCTGGTC
	Glutathione Peroxidase 1	*Gpx1*	NM_008160	F’ GGAGAATGGCAAGAATGAAGR’ TTCGCACTTCTCAAACAATG
	Glutathione Peroxidase 3	*Gpx3*	NM_008161	F’ ACAAGAGAAGTCTAAGACAGACR’ TGTAGTGCATTCAGTTCAAG
	Iodothyronine Deiodinase Type 1	*DIO1*	NM_007860	F’ GATCTGCTACAAGGGTAAAGR’ TAGTACTTCATCTGGGAACAC
	Iodothyronine Deiodinase Type 2	*DIO2*	NM_010050	F’ CAGTCTTTTTCTCCAACTGCR’ CCAGTTTAACCTGTTTGTAGG
	Iodothyronine Deiodinase Type 3	*DIO3*	NM_172119	F’ AAGAAAGTCAAAGGTTGTGGR’ AAAACGTACAAAAGGGAGTC
	Selenoprotein F	*SelenoF*	NM_053102	F’ CTACAGATCAAGTATGTTCGAGR’ TATATGCGTTCCAACTTCTC
	Selenoprotein S	*SelenoS*	NM_024439	F’ ACCTGATGTTGTTGTTAGCR’ CTCTTCTTCAAGCTGTCTTAG
	Selenoprotein K	*SelenoK*	NM_019979	F’ TGATTCCAGATACGACGATGR’ CATTTACCTTCCTCATCCAC
	Selenoprotein M	*SelenoM*	NM_053267	F’ GACAGTTGAATCGCCTAAAGR’ TGGTAATTTCGGCTTAACAG
	Selenoprotein T	*SelenoT*	NM_001040396	F’ GTTCCAGATTTGTGTATCCTGR’ GTGTCTATAAATTGGTTGAGGG
	Selenoprotein N	*SelenoN*	NM_029100	F’ CTTCAAGAAGGTCAACTACCR’ AGCAAGATGGAATGAACAAG
	Selenoprotein P	*SelenoP*	NM_001042613	F’ ATGACTTCCTCATCTATGACAGR’ GAGGTCACAGTTTACAGAAG
House Keepers	Beta-Actin	*Actb*	NM_007393	F’ GATGTATGAAGGCTTTGGTCR’ TGTGCACTTTTATTGGTCTC
	Ubiquitin C	*Ubc*	NM_019639	F’ GAGACGATGCAGATCTTTGR’ ATGTTGTAGTCTGACAGGG
	Hypoxanthine Phosphoribosyltransferase 1	*Hprt1*	NM_013556	F’ AGGGATTTGAATCACGTTTGR’ TTTACTGGCAACATCAACAG
	Topoisomerase 1	*Top1*	NM_009408	F’ GAAATTCCTAGAGCATAAAGGGR’ GGACTCAGCTTCATAACTTTAC
	18S Ribosomal RNA	*Rn18s*	NM_003278	F’ CAGTTATGGTTCCTTTGGTCR’ TTATCTAGAGTCACCAAGCC
